# Mutational landscape of adult ETP-ALL

**DOI:** 10.18632/oncotarget.1106

**Published:** 2013-06-23

**Authors:** Martin Neumann, Philipp A. Greif, Claudia D. Baldus

**Affiliations:** Charité, University Hospital Berlin, Campus Benjamin Franklin, Department of Hematology and Oncology, Berlin, Germany; Department of Internal Medicine 3, Ludwig-Maximilians-Universität (LMU), Munich, Germany, German Cancer Consortium (DKTK), and German Cancer Research Center (DKFZ), Heidelberg, Germany; Charité, University Hospital Berlin, Campus Benjamin Franklin, Department of Hematology and Oncology, Berlin, Germany

T-lineage acute lymphoblastic leukemia (T-ALL) accounts for approximately 25% of adult acute lymphoblastic leukemia. Improving therapy is a major challenge as outcome in adult T-ALL remains unsatisfactory for the majority of patients [1]. The subgroup of early T-cell precursor (ETP) ALL recently gained great interest [2] due to the fact that this subgroup in pediatric T-ALL patients was clinically characterized by an especially poor outcome and molecularly defined by a distinct gene expression signature and an immature immunophenotype [3]. The gene expression signature displays an expression profile similar to that of normal T cell progenitors that immigrate from the bone marrow to the thymus. In addition to properties of T cell development, gene expression profile and immunophenotype of ETP-ALL also show features of an early myeloid fate. Thus, ETP-ALL might serve as model for a stem cell leukemia at the intersection of the myeloid and lymphoid lineages. The homogeneity of ETP-ALL with respect to gene expression, immunophenotype, and clinical outcome is however not reflected by a common genetic event; in contrast, a comprehensive genomic analysis revealed a highly heterogeneous mutational spectrum of ETP-ALL in pediatric patients [4]. Similarities to myeloid malignancies were found and mutated target genes serving as potentially druggable lesions have recurrently been identified (e.g. cytokine receptors or JAK signalling).

In our recent study we further investigated these aspects for ETP-ALL in adult patients [5]. This analysis included whole exome sequencing of five ETP-ALLs and initially resulted in the identification of only two recurrently mutated genes. Interestingly, the affected genes, *DNMT3A* and *FAT3*, have not been previously been reported in pediatric ETP-ALL patients. The overall mutational spectrum of adult ETP-ALL also comprised genes that have shown to be mutated in T-ALL (*ETV6*, *NOTCH1, DNM2*) as well as in myeloid malignancies (*NRAS*, *JAK1*, *DNMT3A*).

The heterogeneity of genetic alterations is not only observed in ETP-ALL, but also in other hematologic malignancies like acute myeloid leukemia [6]. Thus, supposed homogenous disease entities share only to minor degree common genetic lesions. The generation of genomic data opens the opportunity to classify disease entities on an additional level, which may provide the basis for the development of targeted therapy. For the rare subgroup of ETP-ALL, the collected data are obviously not sufficient to establish definite molecular classifiers. But these data can build in the model of the myeloid/lymphoid leukemic intersection and the identification of recurrent mutations might unravel potential targets relevant to stem cell like leukemias. In our work, we were able to identify over 60% of the ETP-ALL patients with a mutation in one of the three genes: *FLT3*, *DNMT3A*, and *NOTCH1*. Thus, based on these findings, molecularly defined leukemic subgroups can potentially be targeted by new treatment approaches altering these pathways: demethylating agents, kinase inhibitors, and gamma secretase inhibitors.

In addition to these already recognized alterations, novel recurrent mutations were found in *FAT1*, *FAT3*, and *MLL2* in a high percentage of adult ETP-ALL patients. These data might increase the number of patients, for whom specific treatment approaches can be explored in the future. For *FAT1*, the connection to the WNT pathway and possible ways to antagonize the activation has been discussed in a recent work of Morris and colleagues [7].

Another finding of our analysis was the age dependent occurrence of specific mutations. There were several mutations in adult patients that had not been discovered in pediatric patients such as *DNMT3A*, *FAT1*, *FAT3*, and *MLL2*. Of note, we found *DNMT3A* mutations in 16% of adult ETP-ALL patients, consistent with recent discoveries of frequent *DNMT3A* mutations not only in ETP-ALL, but also in T-ALL [8]. We observed a clear age dependency with the occurrence of *DNMT3A* mutations mainly in older patients. In contrast, other mutations like the histone modifiers *EZH2* and *SUZ12* were significantly less frequent in adult than in pediatric patients. Together with data from other studies, which show a higher mutational burden in older patients and the occurrence of mutations in epigenetic regulators, e.g. *TET2* in elderly healthy persons, this age dependency points to the requirement of age adapted therapies. This does not only include the dose modification of existing therapies and the consideration of comorbidities, but in particular the identification of molecular targets within a different genomic background. Keeping in mind that the majority of our recent data were collected in patients aged 60 years and younger, many more patients over the age of 60 years will have to be studied in detail to unravel genetic alterations in the elderly.

In summary, next generation sequencing allowed to explore the mutational spectrum of adult ETP-ALL and led to the identification of novel recurrent mutations and potentially druggable targets. The enormous challenge yet lies ahead: the development of targeted therapies and the implementation of a routine screening for these mutations resulting in consecutive tailored therapy based on the genetic lesions.
